# Radiant Motherhood

**Published:** 1920-11-20

**Authors:** Marie Carmichael Stopes


The Editor's Letter Box—(*continued*).

### "Radiant Motherhood."

*To the Editor of* The Hospital.

Sir,—In your number of November 6 I have to thank you, in reviewing my new book *Radiant Motherhood*, for recognising that I am "intensely in earnest." At the same time your reviewer remarks that I fall into "grotesque errors" now and then.

It chances that I have received recently private letters of warm appreciation from a man whom many consider the greatest physiologist in Europe, and all would agree is among the two or three greatest, who says of my books:

"There is scarcely anything in what you have written that I am inclined to disagree with"; and "the accuracy of your physiological statements is surprising."

I might also add that *Radiant Motherhood* was read and approved in detail in manuscript by leading medical and scientific experts. I feel, therefore, great surprise that anything in it can be described as "grotesque error," and I must ask you to publish this letter, and at the same time to publish an explicit statement from your reviewer of all such errors in the book, quoting the exact page and reference.

I trust that, as space in your paper is so valuable, you will rule out of your reviewer's reply all items upon which experts may legitimately differ.—I remain, yours faithfully,

Marie Carmichael Stopes.

